# Microbiome, Transcriptome, and Metabolomic Analyses Revealed the Mechanism of Immune Response to Diarrhea in Rabbits Fed Antibiotic-Free Diets

**DOI:** 10.3389/fmicb.2022.888984

**Published:** 2022-07-06

**Authors:** Jie Wang, Huimei Fan, Siqi Xia, Jiahao Shao, Tao Tang, Li Chen, Xue Bai, Wenqiang Sun, Xianbo Jia, Shiyi Chen, Songjia Lai

**Affiliations:** College of Animal Science and Technology, Sichuan Agricultural University, Chengdu, China

**Keywords:** antibiotic-free diet, wean rabbit, gut microbiota, transcriptome, metabolites

## Abstract

In this study, diarrhea was induced in rabbits by feeding them antibiotic-free feed. The gut provides important defense against the barriers of the body, of which the duodenum is an important part to help digest food and absorb nutrients. However, the mechanisms underlying the roles of the gut microbiome and fecal metabolome in rabbit diarrhea caused by feeding an antibiotic-free diet have not been characterized. Recently, only a single study has been conducted to further characterize the antibiotic-free feed additives that caused diarrhea in weaned rabbits. The multi-omics techniques, including 16S rRNA sequencing, transcriptome sequencing, and LC-MS analysis, were combined to analyze the gut microbial compositions and functions. They also determined the fecal metabolomic profiles of diarrhea in rabbits caused by feeding antibiotic-free feed. The results showed that the liver, duodenal, and sacculus rotundus tissues of diarrhea rabbits were diseased, the composition of intestinal microbes was significantly changed, the diversity of intestinal microbes was decreased, and the distribution of intestinal microbe groups was changed. Functional analysis based on the cluster of GO and KEGG annotations suggested that two functional GO categories belonged to the metabolism cluster, and five KEGG pathways related to the metabolic pathways were significantly enriched in diarrhea rabbits. Moreover, real-time quantitative PCR (RT-qPCR) was used to verify the significant expression of genes related to diarrhea. Metabolomics profiling identified 432 significantly differently abundant metabolites in diarrhea rabbits, including amino acids and their derivatives. These amino acids were enriched in the tryptophan metabolic pathway. In addition, the functional correlation analysis showed that some altered gut microbiota families, such as *Parasutterella*, significantly correlated with alterations in fecal metabolites. Collectively, the results suggested that altered gut microbiota was associated with diarrhea caused by antibiotic-free feed additives in weaned rabbit pathogenesis.

## Introduction

In animal production, antibiotics have been used to maintain intestinal health, promote growth performance, and prevent disease. The mechanism of action of antibiotics is mainly concentrated in the gut, which contains a large number of bacteria. Therefore, antibiotics also play an essential role in the prevention of intestinal bacterial diseases (Dibner and Richards, 2005b; Grave et al., 2006; Shang et al., 2020). However, due to the long-term use of antibiotics, the genetic components (bacteriophages, plasmids, naked DNA, or transposons) in animals are repositioned, and the chromosomal sequence in the bacteria is mutated, which leads to a decrease in the total number of intestinal bacteria. In addition, the drug residue of antibiotics and the number of drug-resistant bacteria increased, posing a serious threat to human health (Dibner and Richards, 2005a; Yang et al., 2019; Haque et al., 2020). Consequently, many countries, particularly in Europe, have banned the use of antibiotic growth promoters (AGP) in animal diets (Castanon, 2007). The in-feed antibiotics that were used as growth promoters have been prohibited in China from the beginning of 2020. This increased the pressure to develop safe and effective strategies that can maintain intestinal health and performance in animals (Shang et al., 2020).

Notably, the removal of antibiotics has led to a series of problems, such as increased incidence of intestinal diseases and decreased growth performance (Dibner and Richards, 2005a; Dai et al., 2020). Previous studies have shown that mortality and morbidity of weaned piglets increased after antibiotics were prohibited on Danish pig farms (Wierup, 2001). In the poultry industry, there have been serious problems, such as poor production efficiency, overgrowth of bacteria in the small intestine, poor nutrition absorption, and necrotizing colitis in the chicken population, which are considered to be the major threat factors to the poultry industry (Haque et al., 2020; Jha et al., 2020). Diarrhea is one of the digestive system diseases of young rabbits, accounting for 70% of all diseases, which can further lead to low immunity, disturbance in intestinal microbiota, increased mortality, and serious restriction to the development of the rabbit industry (Chen et al., 2017; Liu et al., 2019).

The duodenum is not only the terminal site for nutrient digestion and absorption but also intimately interacts with a diverse community of intestinal antigens and bacteria to influence gut and whole-body health (Wu, 2022). The metabolism of intestinal content, microorganisms, and the host immune system exchange metabolites in the intestinal cavity and mucosal surface. The interaction between the gut microbiota and the immune metabolism of the host has a profound impact on the host's health (Duan et al., 2021). Although most studies suggest that diarrhea is caused by pathogenic microbial organisms in the gut, there is limited information on the intestinal microbial composition of individuals with diarrhea (Altomare et al., 2021). In terms of metabolomics, some studies have found metabolites associated with diarrhea in different intestinal segments. Nevertheless, further studies are required to elucidate the pathogenesis of diarrhea (Tang et al., 2021). In practice, it is still a challenge and one of the research hotspots to elucidate the biological mechanism of diarrhea in young rabbits fed with anti-resistant diets.

In conclusion, it is time to recognize the central role that gut microbes and intestinal metabolites play in diarrhea. Therefore, this research performed multi-omics studies combined with 16S ribosomal RNA (rRNA) gene sequencing using the contents of duodenum samples. At the same time, it also conducted untargeted liquid chromatography-mass spectrometry (LC-MS) using contents and tissue samples from six diarrhea rabbits and six healthy controls with gene expression profiling from the Gene Expression Omnibus (GEO) database to characterize the gut microbial community and contents of the metabolic profiles. An integrated analysis of the microbiome, metabolome, and transcriptome was also performed. -Together, these results may ultimately provide a basis for further understanding of the pathogenesis and development of novel therapeutic strategies for diarrhea induced by feeding anti-resistant diets in rabbits, as well as the microbial–gene–metabolic axis in the duodenum.

## Materials and Methods

### Animals and Feeding Strategy

The Zhongtian Township Comprehensive Service Center in Sichuan Province selected two hundred 35-day-old Hyplus female rabbits. Newborn rabbits were fed a diet free of antibiotic additives after weaning. All the rabbits were raised under standard farm conditions and vaccinated regularly. Feed materials and additives were formulated according to Institute National de la Recherche Agronomique (INRA) nutrient requirements, and their composition and nutrient content are presented in Supplementary Table 1. Each rabbit was placed in a clean cage (600 × 600 × 500 mm^3^) in an environmental control room (21–23, 60–75% humidity, illumination for 14 h [60 [x]) and was provided with drinking water freely. During the process of feeding, we found that the rabbits gradually developed diarrhea, even leading to death. As a result, six healthy rabbits were randomly selected as the healthy group, and six rabbits with diarrhea were randomly selected as the diarrhea group. The selection of the diarrhea standard was based on the liver, intestinal, and sacculus rotundus histopathological diagnosis (Chen et al., 2021). The Animal Ethical Committee of Sichuan Agricultural University approved all the experimental procedures (approval number: 20210236).

### Fecal Sample Collection, DNA Extraction, and Metabolite Extraction

After the young rabbits with diarrhea and normal young rabbits were killed by bloodletting of carotid artery, the abdominal cavity was opened with sterilized medical scissors and tweezers, and duodenal contents were taken into 2 mL cryostorage tubes. Each sample was repeated six times for microbiome and metabolomics analysis. All duodenal contents were repeatedly rinsed with normal saline until no contents remained. Tissues with a length of about 1 cm were placed into a cryotube, and each sample was repeated six times for transcriptome analysis. In short, in addition to three replicates from the duodenal tissue for HE staining, six biological replicates were collected from each rabbit for metabonomics analysis, with a total of 72 fecal samples. Six biologically duplicated tissues were collected from each rabbit for transcriptome analysis, with a total of 72 tissue samples. DNA and RNA were extracted in liquid nitrogen at −80°C. The liver, sacculus rotundus, and duodenal tissues of 12 young rabbits were repeatedly cleaned with normal saline, and tissues with a length of about 1 cm were placed in 1.5 mL tubes containing formalin for the analysis of physiological sections.

### Morphological Section Analysis of Rabbit Intestine

This study selected seven rabbits with diarrhea and three healthy rabbits for liver, intestinal, and sacculus rotundus tissues for morphological section analysis. The intestinal tissue of duodenum specimens was fixed with 4% paraformaldehyde, trimmed, dehydrated, embedded in paraffin, sectioned, dewaxed with xylene, stained with hematoxylin and eosin (HE), dehydrated with alcohol, and sealed with resin. The histological changes of the whole tissue sections were observed under a microscope (HE, bar = 100 μm, power = × 100), and normal areas and obvious lesion areas were recorded with a microscope imaging system (Chen et al., 2021).

### 16S rRNA Sequencing Analysis

Microbial genomic DNA was extracted from duodenal samples of each young rabbit, and total genomic DNA was extracted using cetyltrimethylammonium bromide (CTAB) or sodium dodecyl sulfate (SDS), strictly following the instructions of the manufacturer. DNA concentration and purity were monitored by performing 1% agarose gel electrophoresis (AGE). The sterile water was diluted to 1 ng/μL according to the required DNA concentration. Using the diluted genomic DNA as the template, the V1–V9 region of the bacterial 16S rRNA gene was amplified using (Forward 5'−3': AG; Forward 5'−3': AGAGTTTGATCCTGGCTCAG; Reverse 3'−5': GNTACCTTGTTACGACTTAGTTTGATCCTGGCTCAG; Reverse 3'−5': GNTACCTTGTTACGACTT) specific primers with a barcode by the PCR reaction according to the selection of sequencing regions. After the reaction, the PCR products were mixed in equal quantities, and a 2% AGE was performed. The target strips were recycled for purification (gel recovery kit provided by QIAGEN). The sequencing libraries were generated using the SMRTbell^TM^ Template Prep Kit (PacBio), following the recommendations of the manufacturer. The raw data obtained by sequencing might contain a certain proportion of interference data, which were first filtered to obtain clean data (Su et al., 2021b).

α-Diversity is applied to analyze the complexity of species diversity for a sample through indices, including Observed-species, Chao1, Shannon, and Simpson. All these indices in our samples were calculated using QIIME (v1.9.1) and displayed with R software (v2.15.3)(Oksanen et al., 2017). Two indices were selected to identify community richness, and two indices were used to identify community diversity: the Shannon index (http://www.mothur.org/wiki/Shannon) and the Simpson index (http://www.mothur.org/wiki/Simpson). A principal coordinate analysis (PCoA) was performed to get principal coordinates and visualize them from complex, multidimensional data. The PCoA analysis was performed by the WGCNA package, stat packages, and ggplot2 package in the R software (Bray and Curtis, 1957). The R software was used to analyze the differences between β-diversity indices. Linear discriminant analysis (LDA) and its effect size (LEfSe) were performed to identify the bacterial taxa between healthy and diarrhea groups, which considered an LDA score >4 as significant (Su et al., 2021a).

### Transcriptome Sequence

The total RNA was extracted from the duodenal samples of each young rabbit. RNA integrity was assessed using the RNA Nano 6000 Assay Kit from the Bioanalyzer 2100 system (Agilent Technologies, CA, USA). The RNA was reverse-transcribed into mature messenger RNA (mRNA), and oligonucleotides were used as primers for the PCR reaction. The PCR products were purified, and a database was established. This database included low-quality and high-quality reads; it filtered out low-quality reads and analyzed the high-quality reads. All the downstream analyses were based on clean data with high quality.

Reference genome and gene model annotation files were downloaded directly from the genome website. The index of the reference genome was built using Hisat2 (v2.0.5), and the paired-end clean reads were aligned to the reference genome using Hisat2 (v2.0.5). The mapped reads of each sample were assembled by StringTie (v1.3.3b) in a reference-based approach (Yang et al., 2014). FeatureCounts (v1.5.0-p3) was used to count the number of reads mapped to each gene, and the fragments per kilobase of transcript per million mapped reads (FPKM) of each gene were calculated based on the length of the gene and the read count mapped to this gene (Garber et al., 2011). Before differential gene expression analysis, the read counts for each sequenced library were adjusted by the edgeR program package through one scaling normalized factor. Differential expression analysis of two conditions was performed using the edgeR package in the R software (v3.22.5). A corrected *p*-value of 0.05 and an absolute fold-change of 2 were set as the thresholds for significantly differential expression (Smyth, 2010). The DEGs were subjected to functional enrichment analysis with Gene Ontology (GO) terms, including molecular function, cellular component, and biological process terms, as well as Kyoto Encyclopedia of Genes and Genomes (KEGG) pathway categories; these analyses were performed with the Metascape online tool (Sun et al., 2015). The GO terms and KEGG pathways with a *p*-value of <0.01 were considered to be significantly enriched.

### qRT-PCR Validation of RNA-seq Analysis

To validate the repeatability of the RNA-seq analysis results, seven candidate genes were randomly selected and evaluated by using quantitative real-time PCR (qRT-PCR) (for the primers, see Supplementary Table 1). We performed qRT-PCR in a CFX96 Real-Time PCR Detection System (Bio-RadCo., Hercules, CA, United States) and detected RNA expression using SYBR Green Real-Time PCR Master Mix (Takara Co., Dalian, China). The relative expression levels were calculated using the 2 ^−ΔΔ^Ct method and normalized to those of the reference gene GAPDH.

### Metabolomic Sequencing

The metabolome measurement and pretreatment procedures were based on the protocols followed by Majorbio Bio-Pharm Technology Co., Ltd. (Shanghai, China). These detailed steps were consistent with the published article of the fan. Principal component analysis (PCA) and partial least squares-discriminant analysis (PLS-DA) were performed on metaX (a flexible and comprehensive software for processing metabolomics data). This study applied univariate analysis (*t*-test) to calculate the statistical significance (*p*-value). The metabolites with variable importance of projection (VIP) >1, a *p*-value <0.05, and a fold change (FC) ≥2 or FC ≤ 0.5 were considered to be differential metabolites. Volcano plots were used to filter metabolites of interest based on log2 (fold change) and –log10 (*p*-value) of metabolites. The functions of these metabolites and their metabolic pathways were studied using the KEGG database. The enrichment of metabolic pathways of differential metabolites was performed when the ratio was satisfied by x/n > y/N. On the other hand, the metabolic pathways were considered to be enriched statistically significantly when the *p*-value of the metabolic pathways was <0.05 (Su et al., 2021a).

### Correlation Analysis of Duodenum Intestinal Bacteria, the DEGs, and the DMs

The Pearson correlation analysis was employed to reveal the correlation between the intestinal bacteria and the intestinal immune-related DEGs of the host and tissue duodenal metabolites (DMs) using the Cytoscape software CoNet plug-in, while the correlation coefficient and *p*-value threshold were not set. A *p*-value <0.05 was regarded as statistically significant, a *p*-value <0.01 was regarded as very significant, and a *p*-value <0.001 was regarded as extremely significant. The correlation between intestinal bacteria and DMs was shown by a heat map. A KEGG pathway diagram was used to show the correlation between DMs and inflammatory genes (Altomare et al., 2021).

## Results

### Characteristics of Diarrhea Rabbits Fed an Antibiotic-Free Diet

**Figure 1:**
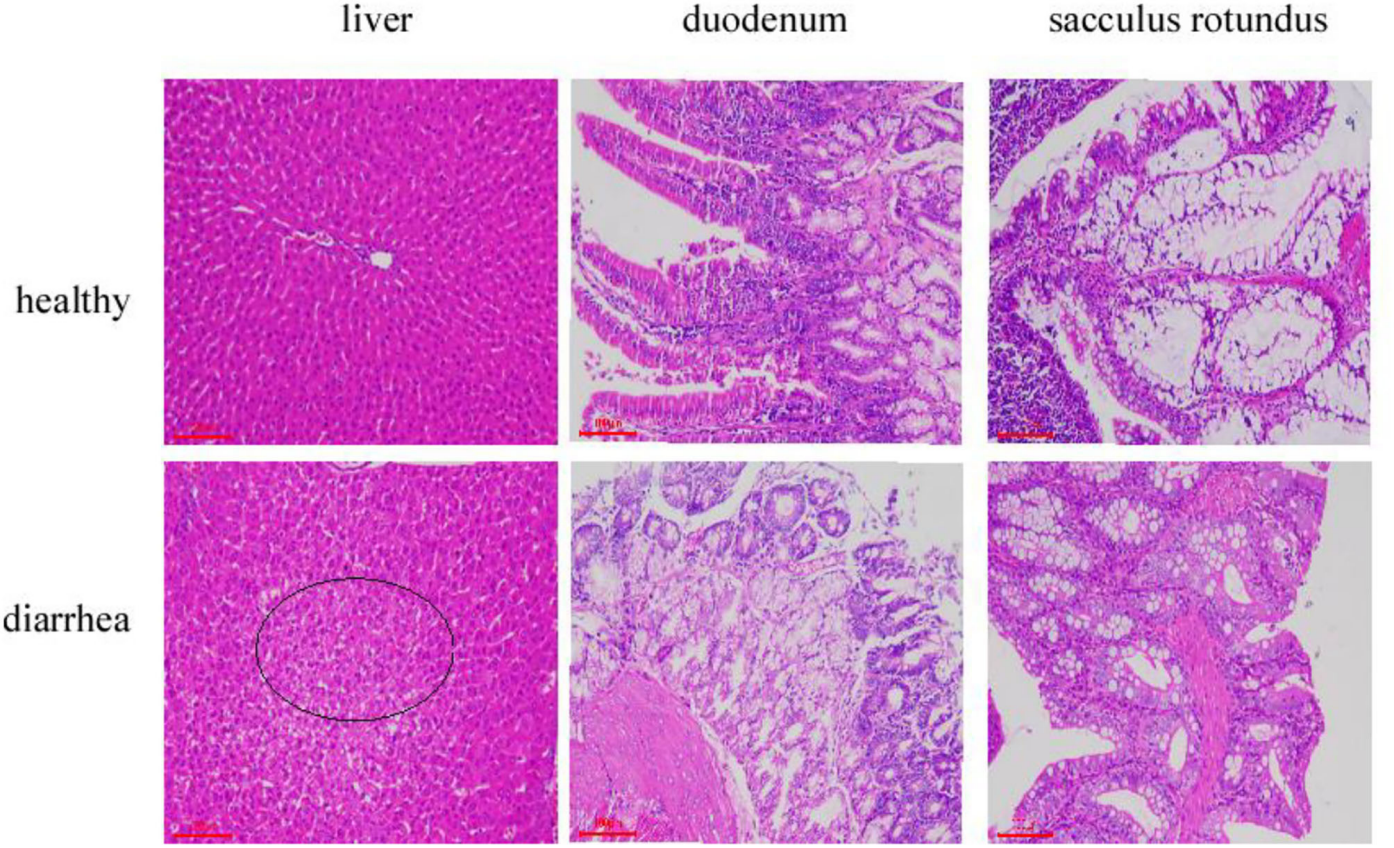
Liver, duodenum, and sacculus tissue samples stained with hematoxylin and eosin (×100) from Hyplus rabbits fed either an antibiotic-free diet or a healthy diet. Black circles indicate pathological features.

Tissue section analysis was used to determine if diarrhea occurred in rabbits fed on the diet without antibiotics. As expected, histopathological examination revealed that the major pathological manifestations of liver injury were sinus congestion and hepatocyte swelling and degeneration. Duodenum has different degrees of necrosis; the intestinal wall necrosis is the most serious; most lymphatic follicles in the number of lymphocytes decreased, followed by mucosal epithelium necrosis, erosion. The epithelial cells of sacculus rotundus were necrotic and exfoliated, the number of lymphocytes in the lower lymphoid tissue decreased, and more tissue cavities were formed. The clinical manifestations indicate diarrhea. All the three tissues and organs in the healthy group were normal (Figure 1).

### Alternative Gut Microbiota Composition in Diarrhea Rabbits

**Figure 2:**
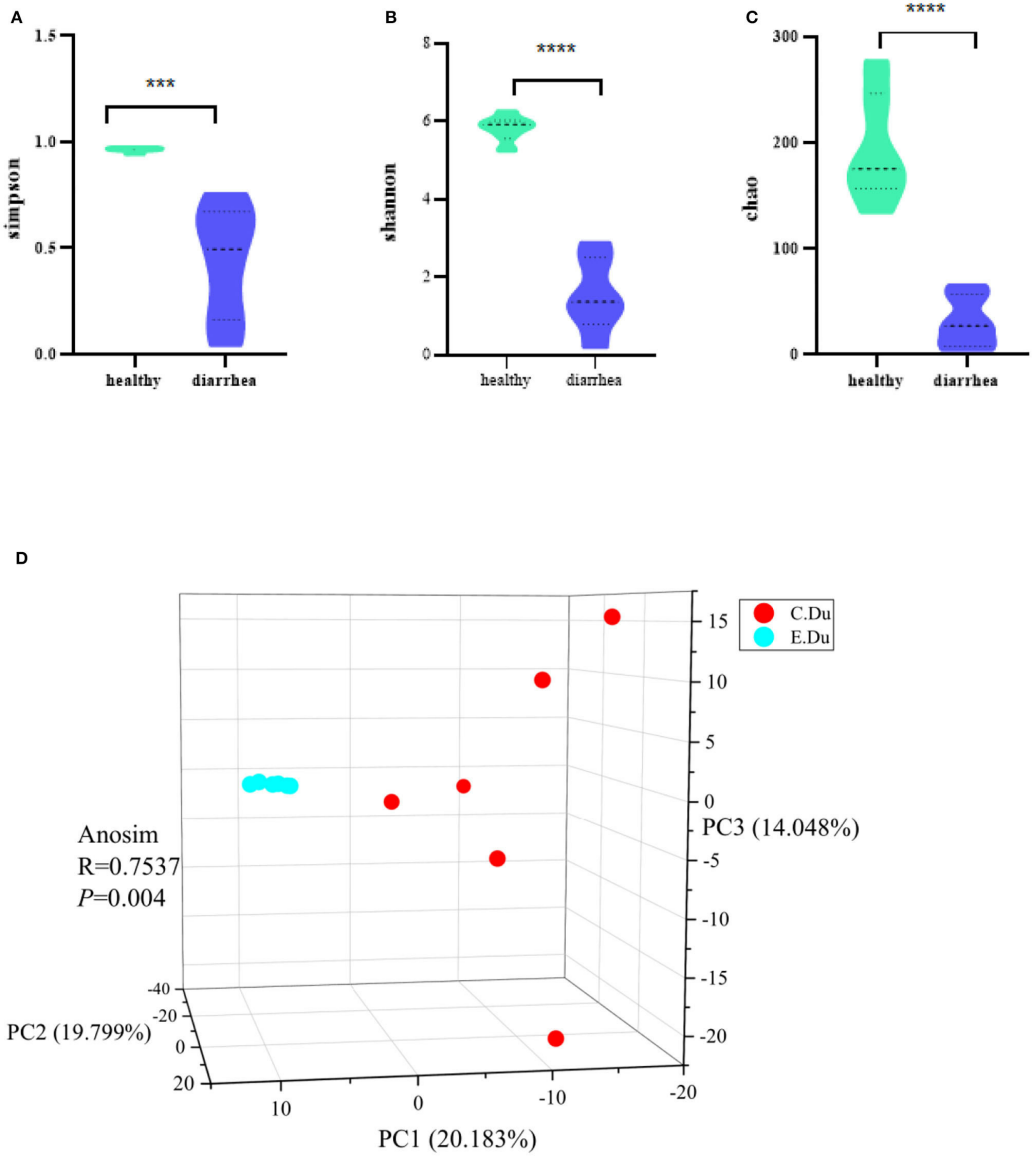
Gut microbial diversity in healthy and diarrhea rabbits. Alpha diversity was evaluated based on the Chao **(A)**, Simpsoneven **(B)**, and Shannon **(C)** indices of the OTU levels. ***P < 0.05, ****P < 0.001. Principal coordinate analysis of beta-diversity was based on the weighted UniFrac **(D)** analyses of the OTU levels. C. Du, healthy; E. Du, diarrhea.

The Chao, Simpsoneven, and Shannon indices were used in this study to estimate the richness, evenness, and diversity of microbial α-diversity, respectively. This research found that α-diversity was significantly reduced in diarrhea when compared to the healthy rabbits (*p* < 0.001 for the Chao richness index, Figure 2A; *p* = 0.046 for the Simpsoneven evenness index, Figure 2B; and *p* < 0.001 for the Shannon diversity index between two groups, Figure 2C; Supplementary Table 2). The results showed that there were significant differences in bacterial diversity in the duodenum between healthy and diarrhea rabbits.

Moreover, the PCoA of weighted UniFrac distances was used to measure the β-diversity in each group. The results showed that the gut microbiota of diarrhea rabbits was significantly different from that of healthy rabbits in both weighted UniFrac distances (ANOSIM R = 0.7537, *p* = 0.004, Figure 2D). Moreover, these results indicated that β-diversity in the diarrhea rabbits was different from that of the healthy rabbits. Therefore, it was concluded that the structural diversity of the gut microbiota was significantly different in diarrhea rabbits as they were fed antibiotic-free feed.

### Altered Composition of the Gut Microbiota in Diarrhea Rabbits

**Figure 3:**
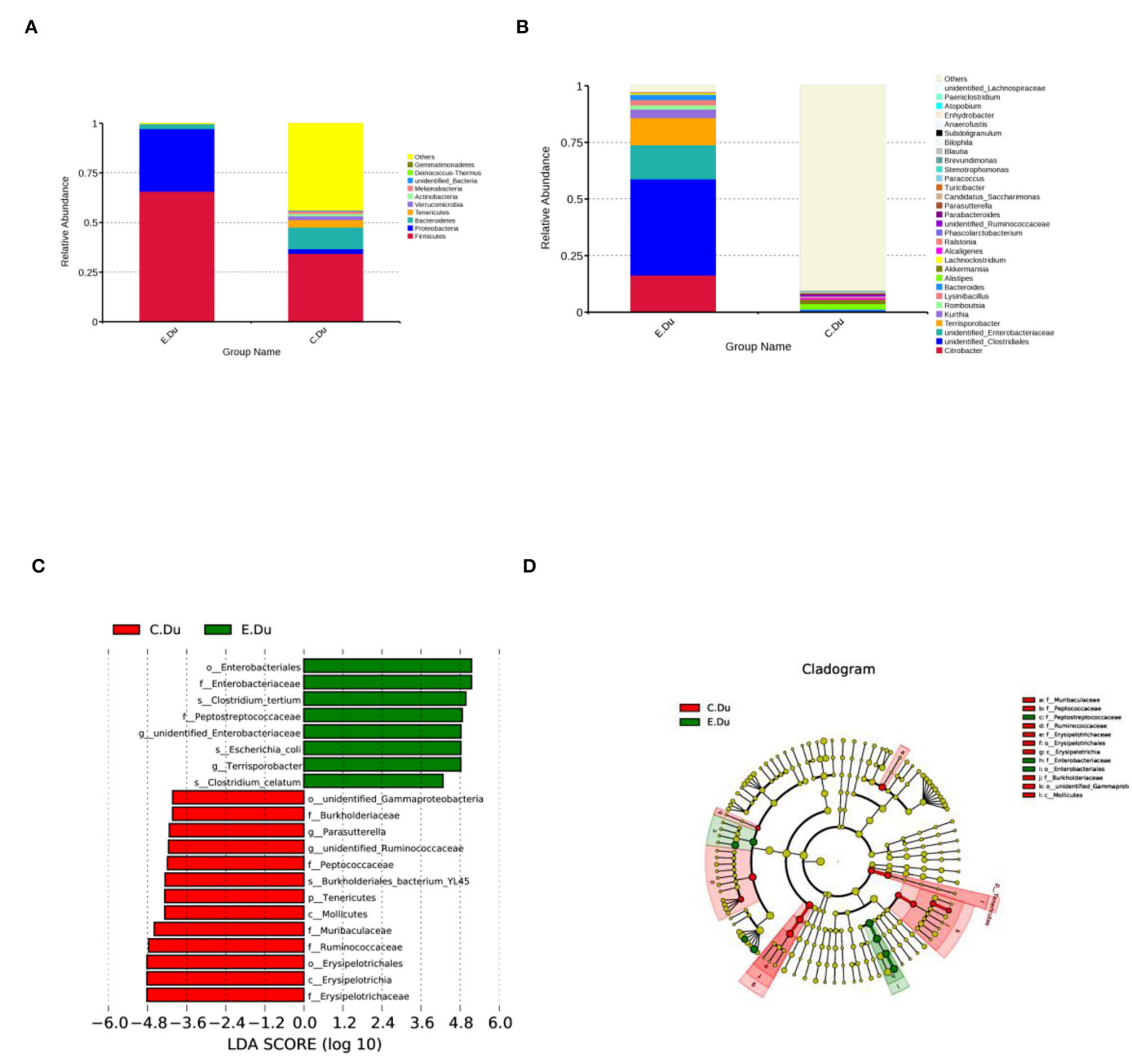
Gut microbiota composition profiles in healthy and diarrhea rabbits. **(A)** Summary of the relative abundances of bacterial genera detected in healthy and diarrhea rabbits. **(B)** Genus-level bacteria were significantly different between the healthy and diarrhea rabbits. Data were shown as relative abundance (%) of the top 10 most abundant genera in each group. Statistical analysis was performed by the Wilcoxon rank-sum test. **(C)** Cladogram generated from the LEfSe analysis indicating the phylogenetic distribution from phylum to genus levels of the microbiota of healthy and diarrhea rabbits. **(D)** Histogram of LDA scores to identify differentially abundant bacterial genera between healthy and diarrhea rabbits (LDA score ≥ 2.5).

As shown in Figure 3A; Supplementary Table 3, the taxonomic analysis indicated that the relative abundance of 11 phyla varied between diarrhea and healthy rabbits. Among these phyla, *Firmicutes* (393.20%) was the predominant phylum in diarrhea rabbits, followed by *Proteobacteria* (189.12%), *Bacteroidetes* (15.19%), and *Tenericutes* (0.23%). In healthy rabbits, the prevalent genera were *Bacteroidetes* (68.93%), *Tenericutes* (22.68%), and *Verrucomicrobia* (11.34%).

Furthermore, Wilcoxon rank-sum tests were performed to compare the differences in fecal bacterial communities between the two groups at the genus level. The results revealed that 12 genera were significantly different between the two groups. Of these discriminatory taxa, *Citrobacter*, unidentified *Clostridiales, Terrisporobacter*, and *Lysinibacillus*, and unidentified *Enterobacteriaceae, Bacteroides*, and *Kurthia* were significantly more abundant in diarrhea rabbits than in the healthy rabbits, whereas *Akkermansia* was significantly more abundant in the healthy rabbits (Figure 3B; Supplementary Table 4).

The linear discriminant analysis effect size was then used to determine whether specific bacterial taxa were differentially enriched in diarrhea rabbits compared to healthy rabbits. Using a logarithmic LDA score cutoff of 2.5, this study identified 20 discriminatory genera as key discriminants (Figure 3C). Several genera, including unidentified *Clostridiale, Enterobacteriales, Enterobacteriaceae, Clostridium tertium, Peptostreptococcaceae, Terrisporobacter*, and *Clostridium celatum*, were significantly overrepresented in the feces of diarrhea rabbits, whereas the genera *Ruminococcaceae, Muribaculaceae, Erysipelotrichaceae, Erysipelotrichia*, and *Erysipelotrichales* were observed to be enriched in healthy rabbits. A cladogram representing the taxonomic hierarchical structure of the fecal microbiota from phylum to species levels indicated significant differences in phylogenetic distributions between the microbiota of diarrhea and healthy rabbits (Figure 3D). These results showed a remarkable difference in the composition of the fecal microbiota of the duodenum between healthy and diarrhea rabbits.

### Effects of Diarrhea on Different Tissues in Rabbits

**Figure 4:**
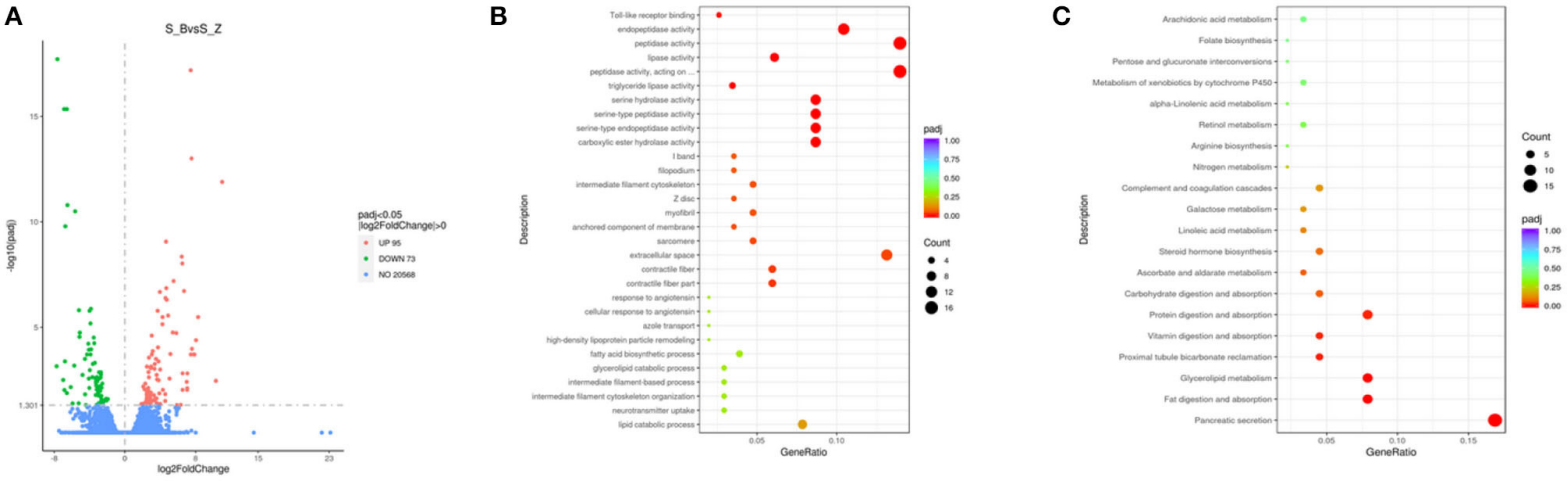
Compared with CG, volcanic plots of DEGs in the duodenum of rabbits in the EG **(A)**; The most enriched GO terms in the intestine of rabbits after diarrhea **(B)**; The KEGG in the duodenum **(C)**. CG, healthy group; EG, diarrhea group.

In the duodenum, 168 DEGs were identified in the EG (Diarrhea group) compared to the CG (Healthy group). Specifically, compared to the EG, 95 genes were u *p*-regulated, and 73 genes were down-regulated (Figure 4A; Supplementary Table 5)

The GO analysis was performed to identify biologically significant differences in functional categories between healthy and diarrhea rabbits. This analysis provided insights into the functional properties of duodenum tissue. As shown in **Figure 6A**, this study found 30 significantly different functional GOs between healthy and diarrhea rabbits. Of these 30 GO categories, five functional GO categories were highly enriched in the diarrhea group, including the fatty acid biosynthetic process and glycerolipid catabolic process (Figure 4B; Supplementary Table 6).

### KEGG Functional Annotation and Analysis

A LEfSe analysis was performed to explore KEGG pathways with significantly different abundances between healthy and diarrhea rabbits (**Figure 6B**). Based on the threshold values, LDA >2.5, *p*-value <0.05, and 20 KEGG pathways, arachidonic acid metabolism, xenobiotic metabolism by cytochrome P450, retinol metabolism, arginine biosynthesis, and pentose and glucuronate interconversions were found to be significantly enriched in diarrhea rabbits (Figure 4C; Supplementary Table 7).

**Figure 5:**
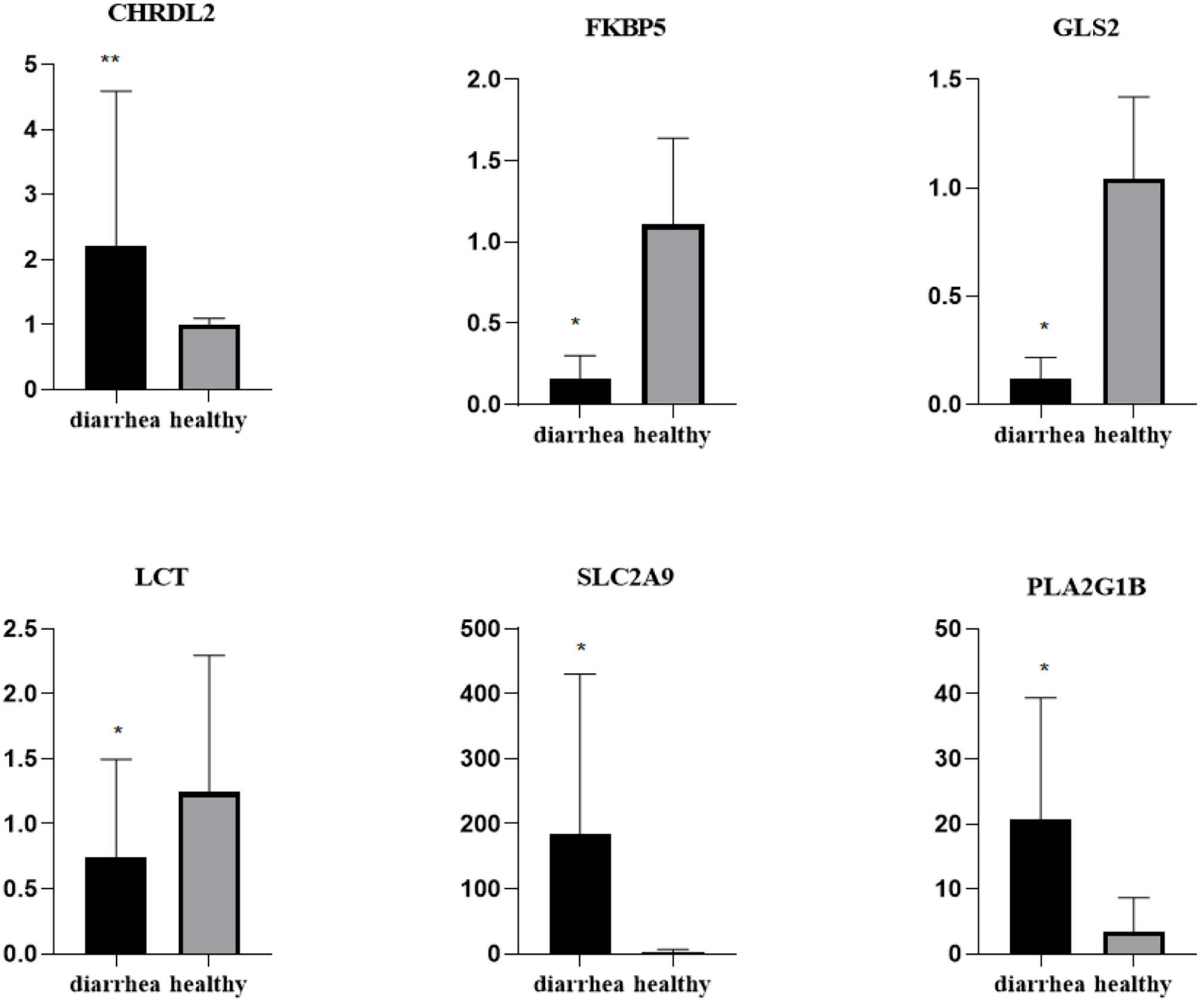
Expression levels of genes CHRDL2, FKBP5, GLS2, LCT, SLC2A9, and PLA2G1B validated by RT-qPCR. The GADPH gene was used as an internal control, and the relative quantity of gene expression (fold change) of each gene was calculated with the comparative 2^−ΔΔCT^ method. Values (RT-qPCR) shown are mean with SD. *P < 0.05; **P < 0.01.

To validate the repeatability of the identified DEGs from transcriptome sequencing, this study randomly selected seven genes, namely, CHRDL2, RFBP5, GLS2, LCT, PLA2G1B, SLC2A9, and AKRIB10 for RT-qPCR analysis (Figure 5; Table 1). According to the findings, these genes were significantly differentially expressed and were consistently upregulated or downregulated with the gene expression changes based on RNA-sequencing analysis, which indicated that the RNA-sequencing data were reliable.

**Table 1:** Validation of selected RNA-seq-based gene expression data by qRT-PCR.

**Tissue**	**Gene**	**Healthy vs. diarrhea**	
		**RNA-seq(log2fold change)**	**qRT-PCR(2 –^ΔΔ^*Ct*)**
Duodenum	AKR1B10	−2.751	0.198
	CHRDL2	8.291	2.215
	FKBP5	3.303	0.159
	GLS2	−3.935	0.116
	LCT	−2.801	0.747
	PLA2G1B	−3.927	2.487
	SLC2A9	6.490	1.834

### Alterations in the Fecal Metabolic Profile of Diarrhea Rabbits

**Figure 6:**
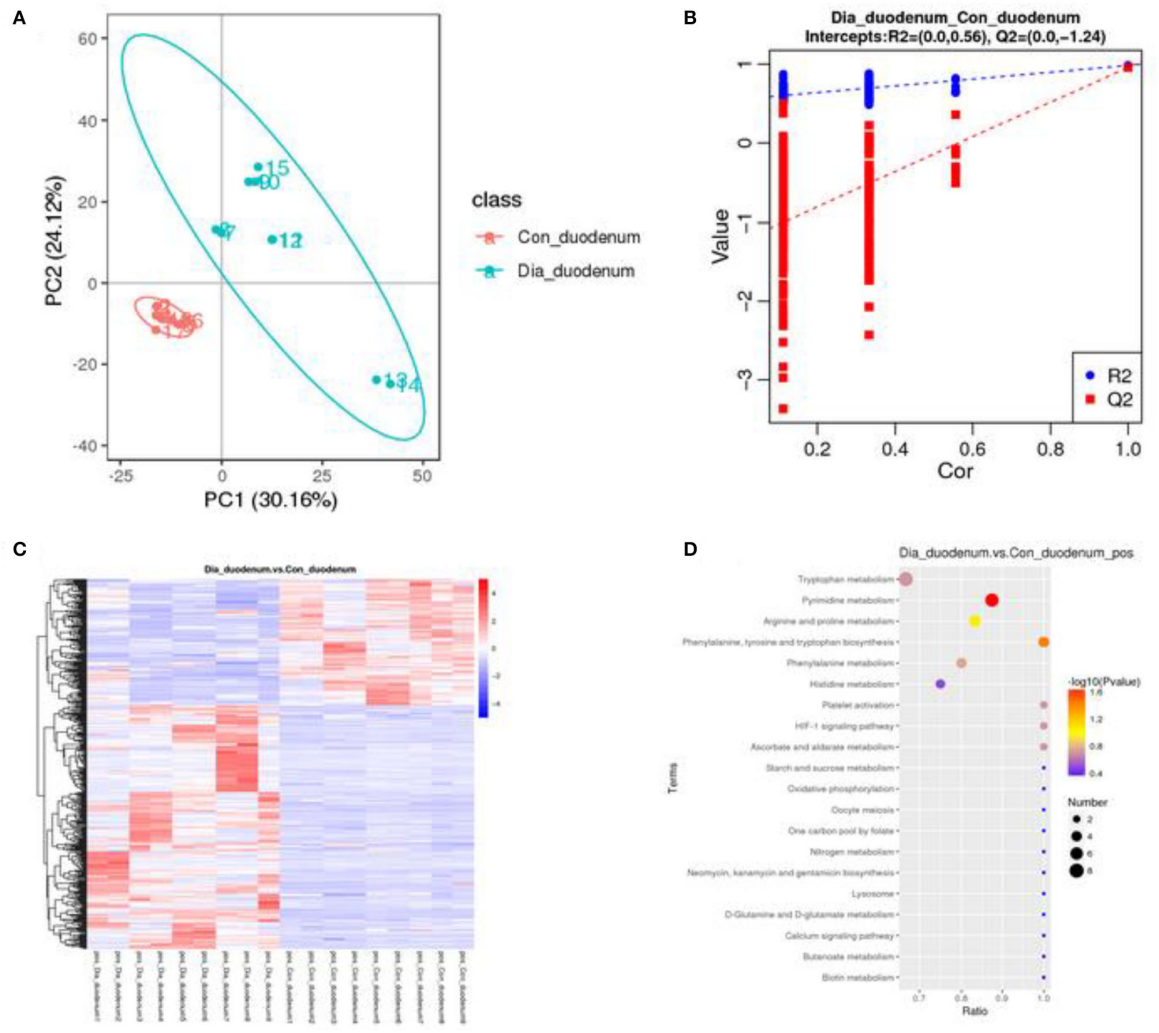
Multivariate statistical analysis of fecal metabolites in healthy and diarrhea rabbits **(A)** PCA and OPLS-DA plots showing the spatial division between healthy and diarrhea rabbits **(B)**, heatmap and KEGG of differences in the metabolites of metabolic pathways in duodenum between the CG and the EG, which was significant **(C,D)**.

A principal component analysis algorithm was used to distinguish the inherent trends within the metabolic data of healthy and diarrhea rabbits. As shown in Figure 6A, differences were observed between the two groups, which indicated inherent metabolic differences between them. To further identify metabolites that discriminated between healthy and diarrhea rabbits, an orthogonal partial least squares-discriminant analysis (OPLS-DA) model was constructed. The OPLS-DA score plot showed clear discrimination between the two groups (R2 = 0.56, Q2 = −1.24), which suggested that the model was predictive and reliable, and that differences in the abundance of the metabolites between healthy and diarrhea rabbits were highly significant (Figure 6B). Differential metabolites that were statistically significant among the two groups were those with a VIP value >1 and a *p*-value <0.05.

### Metabolic Variation Analysis in Diarrhea Rabbits

Metabolites with VIP scores >1 in the multivariate model of OPLS-DA and a *p*-value <0.05 were considered to be potential metabolic biomarkers. A total of 1,096 potential metabolic biomarkers and 432 significant differentially abundant metabolites were identified between healthy and diarrhea rabbits. A heatmap was constructed to visualize these 432 significant differentially abundant metabolites (Figure 6C). Overall, 284 and 148 metabolites were significantly higher and lower in diarrhea rabbits, respectively (Table 2). Most of the differentially abundant metabolites were amino acids and their derivatives. Several amino acids, such as L-tryptophan, acetyl-L-carnitine, N-acetylvaline, N6, N6-trimethyl-L-lysine, N-acetyl-L-tyrosine, and anthranilic acid, were among the upregulated metabolites in diarrhea rabbits. KEGG analysis of the significant differential metabolites was performed to investigate the metabolic mechanisms that were affected by an antibiotic-free diet in weaned rabbits with diarrhea. The enrichment analysis results (Figure 6D; Supplementary Table 8) displayed that metabolic pathways, such as phenylalanine, tyrosine, and tryptophan biosynthesis metabolism, were significantly affected (*p* < 0.05) by changing the healthy and diarrhea rabbit groups.

**Table 2:** Significant metabolites among the two groups.

**Pairwise comparison**	**Metabolite**	**Pvalue**	**VIP**	**Up.down**
Healthy vs. diarrhea	L-Tryptophan	1.88E-14	1.880814185	Up
	Hydroquinone	7.67E-13	1.862381203	Up
	5-allyl-4,6-dimethyl-2-oxo-1,2-dihydropyridine-3-carbonitrile	4.59E-12	1.885380869	Up
	3-methyl-5-phenylpyridazine	5.03E-12	1.884557464	Up
	Scopoletin	1.77E-10	1.866099354	Up
	methyl isoquinoline-3-carboxylate	3.71E-10	1.855332731	Down
	4-Methyl-5-thiazoleethanol	1.26E-09	1.871839618	Down
	Oleanolic acid	1.60E-09	1.83180313	Down
	4-hydroxy-3-(3-methylbut-2-en-1-yl)benzoic acid	6.37E-09	1.821850844	Down
	N-Acetylvaline	9.00E-09	1.822929575	Up
	DL-Stachydrine	1.06E-08	1.803461352	Down
	N6,N6,N6-Trimethyl-L-lysine	1.38E-08	1.782552483	Up
	4-Aminoindole	2.05E-08	1.775952478	Up
	2,3,4,9-Tetrahydro-1H-β-carboline-3-carboxylic acid	2.69E-08	1.76887694	Up
	DL-2-(acetylamino)-3-phenylpropanoic acid	4.39E-08	1.752955133	Up
	(±)15-HETE	4.81E-08	1.808713402	Up
	Isoferulic acid	6.40E-08	1.768174331	Up
	Deoxycorticosterone 21-glucoside	6.45E-08	1.790367317	Up
	Xanthurenic acid	8.61E-08	1.763278641	Up
	Cortisone	8.76E-08	1.801736274	Up
	Robenidine	1.21E-07	1.806415302	Down
	Artemisinin	1.29E-07	1.830240633	Up
	Menaquinone	1.44E-07	1.731674386	Up
	Pilocarpine	1.48E-07	1.789127419	Up
	8-Hydroxyquinoline	1.58E-07	1.807953733	Up
	1-benzyl-3-(2-methylphenyl)-3,7-dihydro-1H-purine-2,6-dione	1.64E-07	1.757619139	Up
	N-(5-acetamidopentyl)acetamide	1.95E-07	1.753435866	Up
	ACar 20:4	2.47E-07	1.768885043	Up
	Ecgonine methyl ester	2.52E-07	1.7436808	Up
	Sinapyl aldehyde	3.08E-07	1.733228363	Up
	Glycerophospho-N-palmitoyl ethanolamine	4.33E-07	1.706005819	Down
	Creatine	5.26E-07	1.747118071	Up
	Indole-3-lactic acid	5.85E-07	1.733281562	Up

### Correlation Analysis of Gut Microbiota and Fecal Metabolic Phenotype

**Figure 7:**
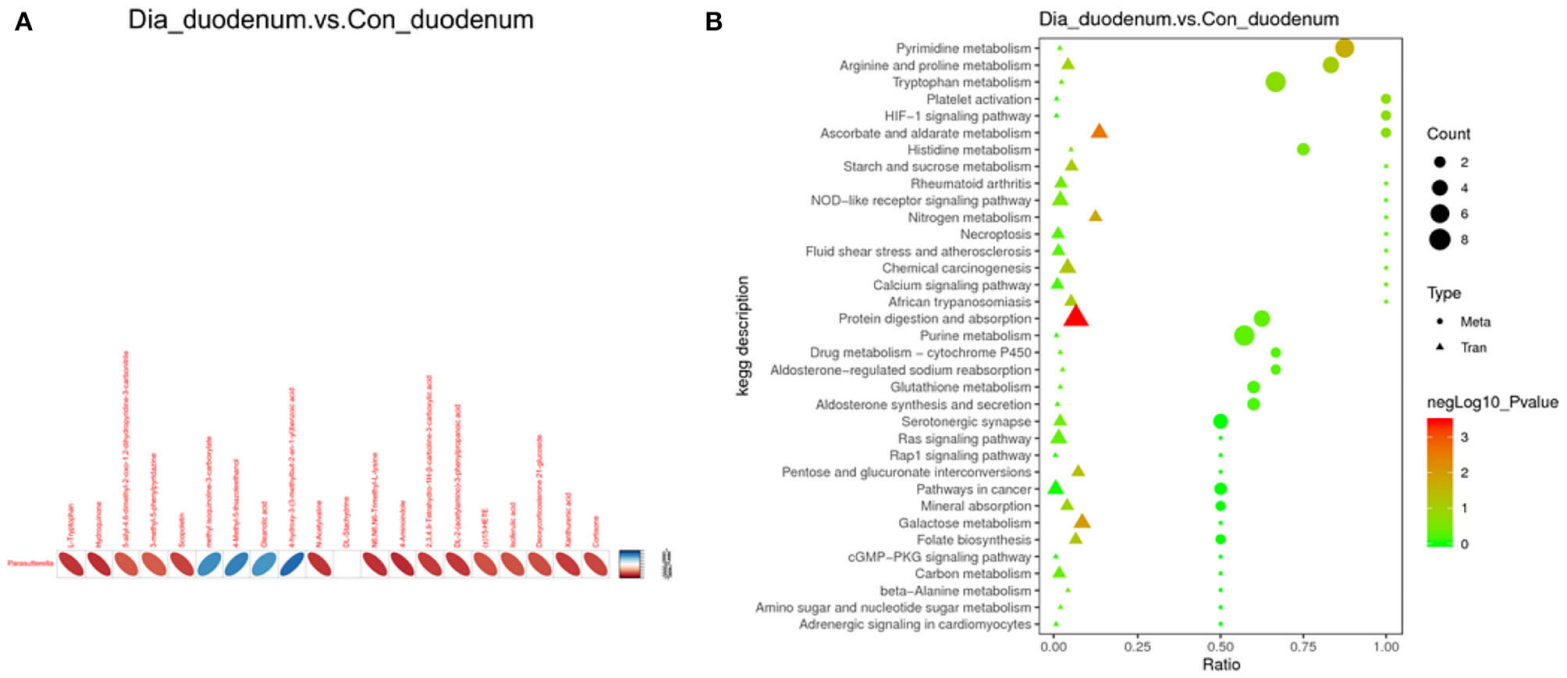
Fecal microbial metabolism and gene expression. Compared with the CG, there was a significant correlation between duodenum intestinal microorganisms and metabolites in the EG **(A)**. Differential genes are involved in the main biochemical pathways and signal transduction pathways **(B)**. Blue, positive correlation; red, negative correlation. CG is the control group; EG is the experimental group; Beta Tran: data type, derived from metabolome or transcriptome analysis.

To reveal the relationships between intestinal microbial, immune, and metabolite parameters, heatmaps were generated by Pearson correlation analysis (Figure 7A; Supplementary Table 9). Among the intestinal bacteria and duodenum metabolism, *Parasutterella* was observed to be negatively correlated with changes in L-anserine, citrinin, protectin D1, calcitriol, and N-acetyl-L-phenylalanine. In the correlation analysis of fecal metabolites and duodenum tissue, protein digestion and absorption were negatively correlated with changes in carboxypeptidase M, trypsin, chymotrypsin, and carboxypeptidase A2 (Figure 7B; Supplementary Table 10).

## Discussion

This study aims to characterize the fecal microbiome of the duodenum in diarrhea rabbits by pathological, integrated 16S rRNA sequencing, gene sequencing, and LC-MS-based metabolomics approaches. The results implicated that the gut microbiota composition and function and fecal metabolic phenotype were significantly different in healthy rabbits compared to those observed in diarrhea rabbits. There are also links between intestinal microflora, microbial metabolites, and protein-related enzymes in tissues.

The pathological results of liver, duodenum, and sacculus rotundus tissue showed that the organs of young rabbits were damaged to varying degrees. Based on the 16S rRNA sequencing results, reduced α- and β-diversity indices and altered gut microbiota composition were observed in diarrhea rabbits compared to healthy rabbits. This suggests that diarrhea may be linked to dynamic changes in the composition of the intestinal microbiota (Huang et al., 2021b). Compared to healthy rabbits, diarrhea rabbits exhibited significantly higher proportions of the phyla Proteobacteria and Firmicutes, whereas significantly lower proportions of Bacteroidetes and Tenericutes. These results were consistent with a previous study (Chang et al., 2020; Zhang et al., 2020; Zhuang et al., 2020; Xi et al., 2021). Firmicutes is the most abundant bacterial phylum of the gut microbiota in diarrhea rabbits and is mainly responsible for decomposing cellulose. It could potentially also have effects on the production of amino acids and their metabolism (Huang et al., 2021a). They are regarded as nutritional targets to prevent or treat diarrhea in diarrhea rabbits (Chen et al., 2020). The phylum Bacteroidetes plays a vital role in digesting carbohydrates and proteins. It also facilitates the maturation of the intestinal immune system (Spence et al., 2006; Sun et al., 2016; Xi et al., 2021). The Proteobacteria, consisting of many Gram-negative bacteria, is the largest bacterial phylum (Das and Chaudhuri, 2003; Howitt et al., 2011). Some members of this phylum are common opportunistic pathogenic bacteria that can cause diarrhea, gastritis, gastrointestinal ulcers, and even death in animals, posing a significant threat to animal health (Nguyen et al., 2011; Fischbach and Malfertheiner, 2018).

Furthermore, this study used RNA-sequencing technology to analyze the effect of diarrhea on the duodenum transcriptome in rabbits. Based on the analysis of the KEGG pathways, diarrhea mainly affects the duodenum, the fatty acid biosynthetic process, and the retinol metabolism process. At the same time, it also changed several genes involved in these metabolic pathways. For example, GLS2 is an enzyme of glutamine, which is an important factor in regulating adaptive immune responses (Scott et al., 2018; Lukey et al., 2019). In this study, the expression of the GLS2 gene was downregulated in the duodenum of diarrhea rabbits. A similar observation was noted in the studies performed on mice. CHI3L2 is secreted by astrocytes and is a secretory protein. It can induce an autoimmune response (Liu et al., 2021). Studies have shown that this gene primarily plays a role in inflammation, and its overexpression is associated with a variety of pathological conditions (Kzhyshkowska et al., 2016). Notably, the upregulation and downregulation of these genes were consistent with the results of real-time fluorescence quantification. Therefore, it was identified that these genes may be one of the causes of diarrhea in weaned rabbits and can be used as targets for the treatment of related diseases.

Due to the importance of microbial-derived metabolites in host health, it is essential to elucidate the metabolic consequences of gastrointestinal dysbiosis and the physiological pathways implicated in specific disease phenotypes (Guard and Suchodolski, 2016). Moreover, four perturbed metabolic pathways were identified in diarrhea rabbits (Supplementary Figure 1). Although only five metabolites are matched to the tryptophan metabolic pathway, this study presumes that this pathway may be perturbed in diarrhea rabbits (Supplementary Table 3). The tryptophan metabolic pathway was also discovered to be the most disrupted in serum samples collected from mice with colitis (Shi et al., 2020). Tryptophan plays an important role in maintaining the balance between gut microbiota and the mucosal immune system. It has been linked to intestinal inflammation and intestinal microbial dysbiosis (Shi et al., 2020; Berlinberg et al., 2021). Moreover, the tryptophan metabolic pathway could be used as a therapeutic target for intestinal dysfunction, inflammatory syndrome, nervous system dysfunction, and other diseases in Wistar rats (Zhao et al., 2019). Certainly, the related metabolites of the tryptophan metabolic pathway need to be quantitatively analyzed by targeted metabolomics, and the role of this pathway in type-2 diabetes mellitus (T2DM) pathogenesis should also be determined in further studies. This study altered the metabolic pathway of phenylalanine in the duodenum and increased its levels. In addition, the levels of aspartate and glutathione were also increased. Phenylalanine regulates the chemotaxis of neutrophils through G-conjugated protein receptors. It is known to have an immunosuppressive effect on the duodenum of rabbits (Sasabe and Suzuki, 2018). Glutathione is found in the duodenum to maintain intestinal homeostasis, and enteroendocrine and energy metabolism (Beaumont and Blachier, 2020). Therefore, this study hypothesized that increased levels of these amino acids could be potential biomarkers for diarrhea in rabbits.

A Pearson correlation analysis was used to analyze the duodenal 16S rRNA sequencing and metabolome results. In particular, host immunity is strongly linked to microbiota composition through poorly understood bi-directional links. Gene expression may be a potential mediator of these links between microbial communities and host functions (Fuess et al., 2021). This research found that after consuming anti-resistant diets, *Parasutterella* was negatively correlated with L-tryptophan, L-lysine, indole, cyanuric acid, and cortisol. A large number of studies have shown that indole and cyanuric acid are metabolites of tryptophan (Berlinberg et al., 2021). The abundance of *Parasutterella*, a genus of *Betaproteobacteria*, has been associated with different host health outcomes, such as inflammatory bowel disease, obesity, diabetes, and fatty liver disease. It has been proven that the colonization of the bacteria can induce changes in the metabolites of the intestinal flora of mice, such as aromatic amino acids, bilirubin, purine, and bile acid derivatives (Ju et al., 2019). These findings imply that the family *Parasutterella* and its associated fecal metabolites may contribute to the inflammation associated with diarrhea.

The results showed that the protein digestion and absorption pathways were the most significant. A large number of studies have shown that protein digestion and absorption are mainly carried out by human intestinal mucosal cells through the carrier protein and γ-glutamine circulation, participating in the intestinal microbial energy metabolism and transforming into a variety of physiologically active substances (Wen et al., 2021). Moreover, carboxypeptidase M, trypsin, chymotrypsin, and carboxypeptidase A2 are enriched in this pathway. These substances are necessary for protein interactions on cell surface membranes. It enhanced the signal transduction of receptors and also enhanced the function of the enzymes, which has the role of resolving inflammatory symptoms. In this study, protein digestion and absorption pathways were negatively correlated with carboxypeptidase M, trypsin, chymotrypsin, and carboxypeptidase A2 (Guimarães et al., 2019; Lewicki et al., 2019). These findings suggest that metabolites in the *Parasutterella* and the tryptophan metabolic pathways in the duodenum are associated with related enzymes in the epithelial tissues. It may be linked to the intestinal barrier and inflammation caused by non-resistant feeding-induced diarrhea.

## Conclusion

**Figure 8:**
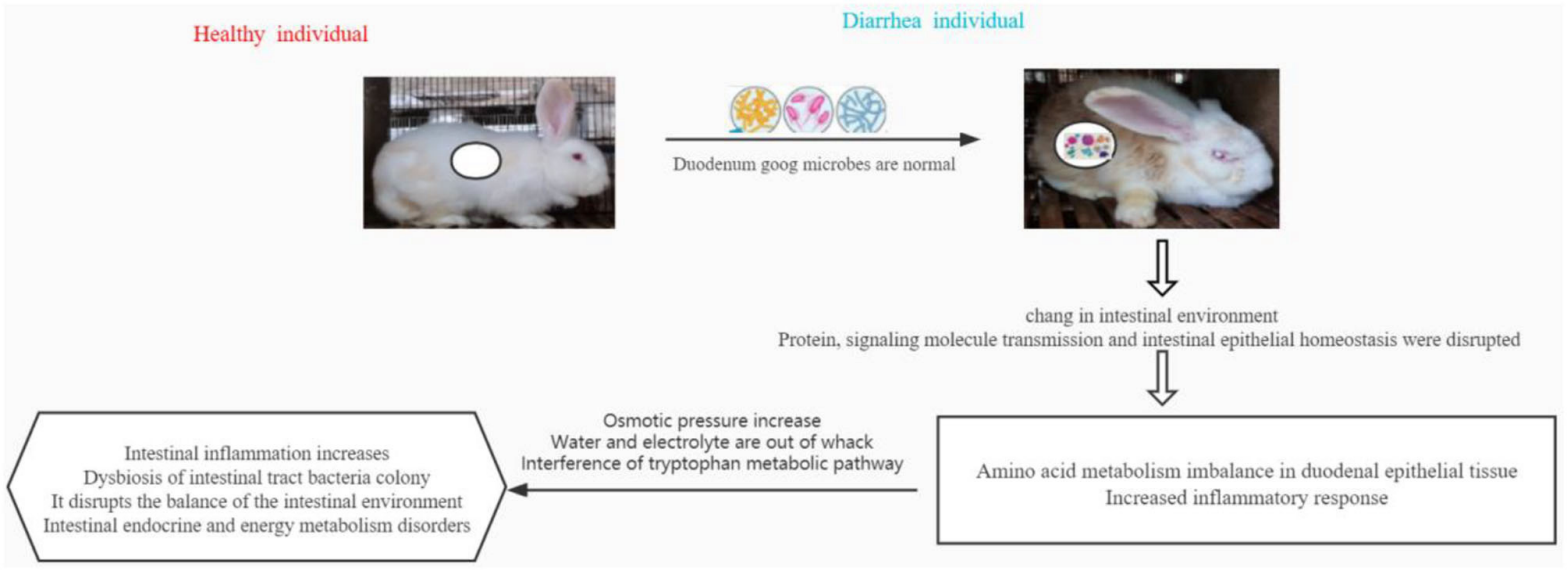
In conclusion, the relative abundance of beneficial microorganisms in the duodenal intestinal tract of weaned diarrhea rabbits changes, which subsequently changes the microbiological environment and immune gene regulation of the duodenal intestinal tract, leading to the imbalance of amino acid metabolism in epithelial tissues and intensified inflammatory response. Further manifestations are elevated osmotic pressure, water and electrolyte imbalance, and endocytosis of intimal proteins, leading to altered pathways of intestinal metabolites. These results mainly show intestinal inflammation and imbalance in the bacterial population of the intestinal tract, disruption of intestinal environmental balance, and disruption of intestinal endocrine and energy metabolism.

In conclusion, the relative abundance of beneficial microorganisms in the duodenal intestinal tract of weaned diarrhea rabbits changes. This leads to another change in the microbiological environment and immune gene regulation of the duodenal intestinal tract. This causes an imbalance of amino acid metabolism in the epithelial tissues and intensifies the inflammatory response. The results further display elevated osmotic pressure, water and electrolyte imbalances, and endocytosis of intimal proteins, leading to altered pathways of intestinal metabolites. These findings mainly include intestinal inflammation, imbalance of intestinal tract bacterial colonies, disruption of intestinal environmental balance, and disruption of enteroendocrine and energy metabolism (Figure 8). This study reports on the potential association of the duodenal gut microbiota with metabolites and genes, thus opening up new possibilities and perspectives for the exploration of reliable diarrhea-related changes.

## Data Availability Statement

The original contributions presented in the study are included in the article/Supplementary Material, further inquiries can be directed to the corresponding author/s. The data presented in the study are deposited in the GSA repository, accession number subPRO014339.

## Ethics Statement

The animal study was reviewed and approved by Sichuan Agricultural University institutional animal care and use committee (Approval No. 20210236).

## Author Contributions

JW, WS, SC, XJ, and SL participated in the conception and design of the experiment. HF, SX, JS, TT, and LC carried out the experiment. HF analyzed the experimental data and drafted the manuscript. HF and XB revised the manuscript. All authors read and approved the manuscript.

## Funding

The current study was funded by High Quality and Characteristic Rabbit Breeding Materials and Method Innovation and New Variety Breeding (breeding research project), the Key R&D Project of Sichuan Province (2021YFYZ0033), and the National Rabbit Industry Technology System Meat Rabbit Variety Improvement (CARS-43-A-2).

## Conflict of Interest

The authors declare that the research was conducted in the absence of any commercial or financial relationships that could be construed as a potential conflict of interest.

## Publisher's Note

All claims expressed in this article are solely those of the authors and do not necessarily represent those of their affiliated organizations, or those of the publisher, the editors and the reviewers. Any product that may be evaluated in this article, or claim that may be made by its manufacturer, is not guaranteed or endorsed by the publisher.
