# Supplementary material for: Microbiome, Transcriptome, and Metabolomic Analyses Revealed the Mechanism of Immune Response to Diarrhea in Rabbits Fed Antibiotic-Free Diets
Source: Front Microbiol. 2022 Jul 6;13:888984. doi: 10.3389/fmicb.2022.888984 (PMC9298518; doi:10.3389/fmicb.2022.888984)
Supplement: Supplementary file 1 [file Table_1.docx]

| Ingredient | SND | | | | | | |
| --- | --- | --- | --- | --- | --- | --- | --- |
|  | Proportion(%) | DE(MJ/kg) | CP(%) | EE(%) | CF(%) | Ca(%) | P(%) |
| Straw powder | 26 | 0.855 | 1.248 | 0.364 | 7.748 | 0.073 | 0.021 |
| Maize | 18 | 2.889 | 1.602 | 0.648 | 0.576 | 0.005 | 0.070 |
| Barley | 20 | 2.808 | 2.040 | 0.34 | 0.860 | 0.020 | 0.093 |
| Bran | 15 | 1.631 | 2.310 | 2.475 | 0.765 | 0.050 | 0.072 |
| Bean cake | 16 | 2.166 | 6.768 | 0.304 | 0.576 | 0.045 | 0.091 |
| Fish meal | 3.5 | 0.552 | 2.047 | 0.196 |  | 0.137 | 0.104 |
| Lard |  |  |  |  |  |  |  |
| Stone powder | 1.0 |  |  |  |  | 0.350 |  |
| Salt | 0.5 |  |  |  |  |  |  |
| Total | 100 | 10.91 | 16.015 | 4.327 | 10.525 | 0.68 | 0.431 |

**Table S1** Composition and nutrient content of the standard normal diet (SND)

Note. DE: digestible [energy](javascript:;); MJ: megajoule; CP: crude protein; EE: ether extract; CF: crude fiber; Ca: calcium; P: phosphorus.
